# Fatigue and Training Load Factors in Volleyball

**DOI:** 10.3390/ijerph191811149

**Published:** 2022-09-06

**Authors:** Damian Pawlik, Dariusz Mroczek

**Affiliations:** Department of Human Motoric Bases, Wroclaw University of Health and Sport Sciences, 51-612 Wroclaw, Poland

**Keywords:** volleyball, female, training load, jumps, acceleration, wellness

## Abstract

The purpose of this article was to determine the internal and external loads experienced by volleyball players in a weekly cycle during the competitive season. Using accelerometers, as well as subjective rating perceived exertion (RPE) and total quality recovery scale (TQR) questionnaires, eleven female athletes were monitored during five days of training sessions over the course of the 2021 season. The data were evaluated for trends during the start period in preparation for the championship tournament. Analysis of the accelerometer data revealed a relationship between RPE and session rating perceived exertion (s-RPE), as well as the number of total accelerations. It was reported that on the training days of the same well-being level, the jump number values were significantly different. The results suggest that monitoring athletes for the number of accelerations can be used to understand the needs of volleyball players and to improve the design of training and recovery days to optimize athletes’ well-being.

## 1. Introduction

Volleyball is characterized by intermittent effort in which high-intensity actions are interspersed with breaks associated with starting the next action or set [1,2,3]. Actions responsible for success in volleyball, such as serving, attacking, and blocking, require repeated high jumps and sudden acceleration and deceleration on a short distance performed by the athlete. Therefore, the important abilities responsible for the foregoing activities will include the strength and explosiveness of the lower extremity muscles [4,5]. Because of that, part of volleyball players’ training focuses on developing jumping skills [2,6]. Volleyball training must address both the demands of the game (technique) and the training load based on volume, intensity, and type of effort [7,8,9]. Monitoring athlete jumps includes assessing the height and number of jumps that athletes make. Relationships between jump height, the number and frequency of jumps, and players’ feelings of fatigue in volleyball are currently being researched [10].

Training Load (TL) in volleyball can be divided into internal training load (ITL) based on players’ subjective feelings and external training load (ETL) related to actual values of kinematic parameters, during a match, practice, or training sessions. In a meta-analysis, Lupo [11] showed that session rating of perceived exertion (s-RPE) constitutes a valuable and relatively quick-to-perform method for monitoring ITL in volleyball players. Confirmation of the accuracy of the s-RPE index in determining ITL can be found not only in volleyball [12,13], but also in other sports [14,15]. Andrade and Debien [1,13] stress the relationship between s-RPE and athlete recovery (Recovery Scale—RS), diagnosed by the Total Quality Recovery (TQR) index. Despite the logical connection between RS and TL, Debien et al. [1] believe that there are additional factors that can cause fatigue in volleyball players. Such factors may include ETL factors. In indoor sports, kinematic analysis is limited due to the lack of ability to use GPS. Video analysis and simple accelerometers that do not impede the game are usually used for kinematic analyses, whereas laboratory tests involve CMJ jump or 20 m run [10,16,17]. An increasing number of load-monitoring devices in team sports have modules that can be used in indoor sports, such as volleyball. Such devices could include a gyroscope and an accelerometer, yet without supporting the orientation of the athlete’s position relative to the body axis, these data appear to be insufficient. Having considered the foregoing, state-of-the-art technology (e.g., Catapult Vector 7) coordinates and calibrates the performance of the above jointly with magnetometer-derived data [18]. Using such an advanced technology, one can easily collect information about Heart Rate (HR), height and number of jumps, acceleration (Accel) and de-acceleration (Decel), or training load (TL).

The primary purpose of this study was to determine the relationship between ETL and ITL indicators. The secondary aim of the study was to estimate the relationship of ETL on the RPE and s-RPE. The search focused on confirming that the height and number of jumps are related to the athletes’ perception of fatigue. It was decided to compile the measured kinematic factors to find relationships between these parameters and athlete fatigue during the training sessions. 

## 2. Materials and Methods

### 2.1. Materials

Fourteen U19 players participated in the study (age: 16–18 years old; height: 172.42 ± 7.3 cm; mass: 63.54 ± 8.36 kg; and BMI: 21.53 ± 2.93) who attended volleyball trainings once a day. Eleven of the fourteen players were included in the performance analysis, while three players did not meet the selection criteria. Only those athletes who participated in each training were included in the result analysis. The study was reviewed and approved by the Senate Committee on Research Ethics of the University School of Physical Education in Wroclaw, Poland. The procedures complied with the Declaration of Helsinki regarding human experimentation.

### 2.2. Methods

The players were monitored during five training days when they were preparing for the play-off phase of the national championships (five training units) (Table 1). During the training cycle, technical and specialized volleyball training was conducted according to the level presented by the players. Anthropometric measurements and motor fitness tests: 20 m run, vertical jump Squat Jump (SJ), Countermovement Jump (CMJ), Countermovement Jump with arm swing (CMJA), and volleyball-specific jumps, such as Spike Jump (AJ) and Block Jump (BJ), were performed on the first day and the day after the cycle. All the results before and after the training camp were collected and tabulated in Table 2.

#### 2.2.1. Physical Performance

All physical parameters were diagnosed after a 30 min warm-up. The warm-up on the first training day and after the training cycle was the same for all the athletes. The athletes waiting for the measurement performed low-intensity ball training to maintain neuromuscular potential after the warm-up. The warm-up included 10 min of general activity (e.g., dynamic stretching, walking, jogging, and exercises), followed by 10 min of dynamic activity with progressive increases in speed and intensity (e.g., skipping, leg, and arm swings), 10 min volleyball skills rally (e.g., overhead and forearm passes, spikes), and three min rest before the first testing session [19].

The athletes performed SJ, CMJ, CMJA [20,21], and volleyball-specific jumps, such as Spike Jump (AJ) and Block Jump (BJ) [22]. All jumps were measured by Optojump equipment (Microgate, Bolzano, Italy). The jumps were performed twice. The better result was included in the analysis.

For the 20-m-sprint, two electronic timing gates (Smart Speed System, Fusion Sport, USA) were positioned at the start line and 20 m finish line. The participants were instructed to begin with their preferred foot forward placed on a line marked on the floor and to run as quickly as possible along the test distance [23]. Each athlete completed three sprints. The first trial sprint was to familiarize oneself with the test and achieve post-activation potential (PAP). Out of the next two runs, the shorter run time was considered for analysis.

#### 2.2.2. Load Data Collection

Before each practice, volleyball players wore markers with player positioning transmitters (Vector S7, positioning 10 Hz Catapult ClearSky, 3D +/− 16G, accelerometer sampled at 1kHz, provided at 100 Hz). The transmitters were previously calibrated for the respective athlete. All kinematic parameters were recorded in the sports hall (Table 3). Inertial Movement Analysis (IMA) is a set of metrics that measures athletes’ micro-movements and direction regardless of unit orientation. Player Load is calculated in the PlayerTek system using the established algorithm. In the case of the PlayerTek system, the accelerometer operates at 400 Hz which is smoothed to 100 Hz with the Player Load calculated as follows:

$$\overset{n}{\underset{i=1}{\sum }}\sqrt{{\left(ax_{i}-ax_{i-1}\right)}^{2}{\left(ay_{i}-ay_{i-1}\right)}^{2}{\left(az_{i}-az_{i-1}\right)}^{2}\;}$$

where *ax_i_*, *ay_i_*, and *az_i_* are the acceleration values in *x*, *y,* and *z* directions respectively, and *i* = 0, …, *n* represents the sampled accelerometer points with *n* + 1 points over the time of the session.

$$Player\;Load=\sqrt{{\left(fwd_{t=i+1}-fwd_{t=i}\right)}^{2}{\left(side_{t=i+1}-siade_{t=i}\right)}^{2}{\left(up_{t=i+1}-up_{t=i}\right)}^{2}}$$

*Fwd* means forward acceleration, side means sideways acceleration, up means upwards acceleration, and *t* means time. 

**Table 3:** Player Load Markers measured during a five-day training period.

Variable	Monday	Tuesday	Wednesday	Thursday	Friday
TRQ Scale (score)	14.86 ± 1.70	16.27 ± 2.01	15.77 ± 2.52	15.68 ± 1.10	15.64 ± 1.43
RPE (Borg Scale)	4.36 ± 0.92	4.09 ± 0.83	4.32 ± 1.23	4.77 ± 0.61	4.09 ± 0.94
s-RPE (Au)	292.36	355.91	721.14	1050.00	470.62
Total Player Load	229.99 ± 25.25	298.54 ± 23.49	229.12 ± 134.03	382.63 ± 62.67	430.27 ± 46.73
AVG Heart Rate	141.18 ± 16.7	141.26 ± 20.29	129.57 ±21.86	132.65 ± 16.09	127.75 ± 14.47
Mean IMA Jump Count Low Band	8.18 ± 5.62	9.73 ± 7.42	6 ± 8.77	10.89 ± 7.87	9.6 ± 6.24
Mean IMA Jump Count Med Band	57.27 ± 15.27	59.64 ± 32.77	26.85 ± 39.01	52.22 ± 31.76	87.3 ± 37.23
Mean IMA Jump Count High Band	17.36 ± 12.66	20.82 ± 21.31	8.95 ± 12.6	17.94 ± 22.57	29.4 ± 27.78
Mean Total Jumps	82.82 ± 20.71	90.18 ± 36.92	41.8 ± 49.59	81.06 ± 45.4	126.3 ± 42.84
Total Jumps Count	911	992	881	1459	1263
IMA Jump Count Low Band	90	107	120	196	96
IMA Jump Count Med Band	630	656	537	940	873
IMA Jump Count High Band	191	229	179	323	294
IMA Accel Low	581	494	776	970	488
IMA Accel Medium	116	155	146	268	99
IMA Accel High	80	59	70	188	98
Total IMA Accel	777	708	992	1426	685
IMA Decel Low	703	788	1075	1394	894
IMA Decel Medium	119	148	163	223	144
IMA Decel High	14	15	25	31	21
Total Decel	836	951	1263	1648	1059
ACC/DEC	0.69	0.52	0.61	0.59	0.46

Total Quality Recovery (TRQ Scale), Rating of Perceived Exertion (RPE), Session Rating of Perceived Exertion (s-RPE), Heart Rate (HR), Inertial Movement Analysis (IMA), Low Band > 20 cm (IMA Jump Count), Med Band 20–40 cm (IMA Jump Count), High Band < 40 cm (IMA Jump Count), acceleration (Accel) and de-acceleration (Decel), IMA Accel/Decel Low > 1.5 m/s^2^, IMA Accel/Decel Med 1.5 m/s^2^–2.5 m/s^2^, IMA Accel/Decel High < 2.5 m/s^2^ training load (TL).

Kinematic parameter data from the transmitters were collected after each training session [15].

Before the training, the athletes completed the TRQ questionnaire about the previous day to determine the level of recovery. The TQR method identifies factors influencing an athlete’s ability to adapt to physical training and structures the regeneration process. The TRQ includes all the important parameters related to the increase in performance (adaptation) or its loss (maladaptation). Two ratios of RPE and s-RPE were determined to determine internal load. The RPE survey was based on the 10-point Borg scale [16,24]. The s-RPE score provided by players was multiplied by training duration (volleyball training minutes) and is presented in AUs to estimate internal TL in accordance with previous methods [15,16,25,26]. The RPE scores were recorded 30 min after the conclusion of training to eliminate bias resulting from the final phase of exercise, as per established methods [15,16,26]. 

#### 2.2.3. Statistical Analysis

The statistical analysis was divided into motor analysis before and after the training cycle. In addition kinematic analysis of the activities on each training day was performed. In motor analysis, intraclass correlation coefficients (ICC) and Cronbach’s alpha reliability coefficient (CA) were calculated to determine reliability between jumps and speed abilities. Inter-variability for each test was measured by the coefficient of variation (CV). The confidence interval (CI) was calculated for the determined mean values of each variable and aimed at marking limiting points within which there was a 95% probability that the sought population means of the variables could be reduced (Table 2). The relationships between s-RPE and the observed variables were searched during the kinematic analysis of the training session actions. Pearson correlation was used for this purpose. A t-test was used to compare the differences in mean values of the motor test variables measured pre- and post-training period. In addition, stepwise multivariate regression was applied in search of a model to explain the s-RPE level, using Statistica 13, SPSS 18 (Table 3). A stepwise multiple regression of the obtained variables was performed in order to find the interdispositions to explain the S-RPE variable (Table 4).

## 3. Results

Table 2 shows the results of motor tests conducted before and after the entire training cycle. It is worth noting the trends toward changes between the studied motor variables. The trends are positive in each parameter, i.e., the athletes generally jumped higher after the training period and had a shorter contact time with the ground than before the applied training cycle. Only 20 m run test had longer values post than the pretest. However, statistically significant differences were noted only in the CMJ jump (Table 2).

### 3.1. Daily Training Load

Table 3 shows the ITL and ETL training parameters for all athletes according to the day of the week. The most taxing day for the players (s-RPE) was Thursday. Thursday was characterized by the highest number of jumps and the highest number of accelerations and decelerations (Table 3). The least stressful training day despite poor recovery (TRQ) was Monday. In contrast, the lowest RPEs were recorded on Tuesday and Friday (4.09 points). It is interesting to note that the difference in the number of jumps between Tuesday and Friday is 271 jumps in favor of Friday. Between Monday and Friday, as many as 352 jumps were in favor of Friday. Nevertheless, looking at the acceleration values, they are close to each other on Monday, Tuesday, and Friday. The extreme difference was 92 accelerations in favor of Monday. Despite the large difference between Tuesday and Friday in the number of jumps, this mean RPE is even at 4.09.

### 3.2. Internal Load and Recovery Scale

**Figure 1:**
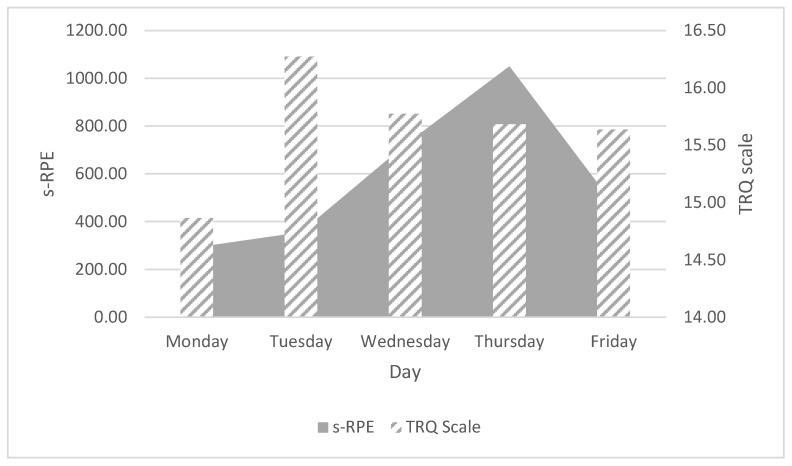
Relations between s-RPE and TRQ scale. Total Quality Recovery (TRQ Scale), Session Rating of Perceived Exertion (s-RPE).

Comparing the ITL parameters, no relationship was observed between TRQ and s-RPE (Figure 1), nor between TRQ and RPE. The best recovery day was Tuesday, after which the TRQ level decreased every day. The lowest s-RPE was recorded on Tuesday while the most difficult training day was Thursday. Both parameters showed no significant association.

### 3.3. Relationships between Internal and External Load/ETL vs. ITL in the Day Comparison

**Figure 2:**
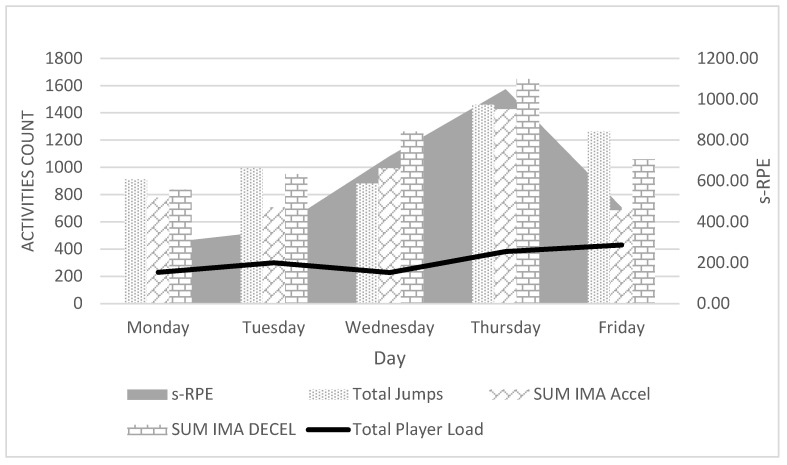
Correlations between s-RPE and ETL factors. Session Rating of Perceived Exertion (s-RPE), External Training Load (ETL), Inertial Movement Analysis Accel/Decel (IMA Accel/Decel).

Figure 2 shows the summary of s-RPE and the total number of jumps (Total Jumps), the sum of accelerations (Sum IMA Accel), and the sum of decelerations (Sum Decel) on each training day. A statistically significant association (*p* = 0.016; Pearson Correlation = 0.944) is demonstrated by the number of accelerations and Sum IMA Decel (*p* = 0.000; PC = 0.996), on a given training day. Interestingly, the number of jumps is not significantly related to s-RPE (*p* = 0.246; PC = 0.638) and Total Player Load (*p* = 0.608; PC = 0.313).

**Figure 3:**
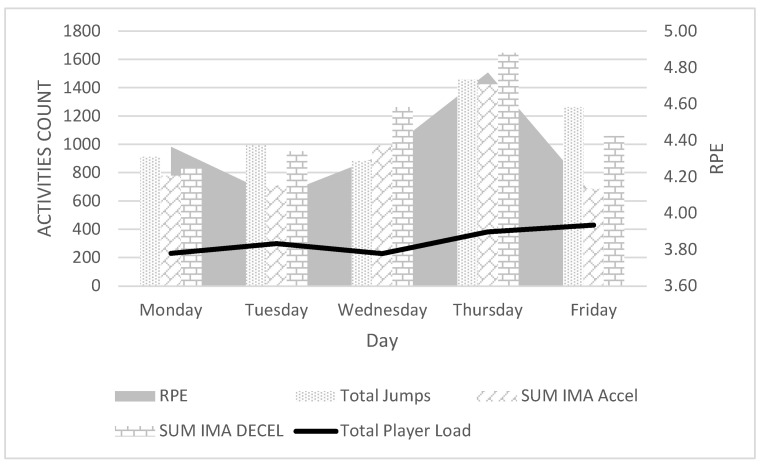
Correlations between RPE and ETL factors. Rating of Perceived Exertion (RPE), External Training Load (ETL), Inertial Movement Analysis Accel/Decel (IMA Accel/Decel).

Having considered the feelings of female RPE athletes, only Sum IMA Accel shows a significantly statistical relationship in this case (*p* = 0.16; PC = 0.943). In contrast, Total Jumps (*p* = 0.382; PC = 508) and Sum IMA Decel (*p* = 0.119; PC = 0.780) show no significant association with RPE level (Figure 3). The discrepancies are particularly evident on Tuesday and Friday, where the s-RPE ratio is low, while the number of jumps and the number of decelerations assume high values (Figure 2). 

### 3.4. Kinematic Predictors without Breakdown by Training Days

When considering all athletes without dividing them by training days, a statistically significant strong relationship was found between RPE and s-RPE (*p* < 0.01) and kinematic parameters Total Player Load, IMA Jump Count Low Band, IMA Accel Low, IMA Accel Medium, Total IMA Accel, IMA Decel Low, Total Decel, Sum Total Accel, Decel and Sum Total Jump, Accel, and Decel (Table 4). The relationships between RPE and s-RPE, as well as and the parameters related to acceleration and braking, are particularly evident. Surprisingly, the jumping parameters, except Jump Count Low Band for jumps, are statistically non-significant. Furthermore, when adding together running and jumping parameters, the index that includes only acceleration and deceleration (Sum Total Accel; Decel) correlates more strongly.

Table 5 compares the players’ accelerations and decelerations to the height and number of jumps, excluding the libero. The results indicate that both the total number of jumps (Total Jumps Count), the mean number of jumps (Mean Total Jumps), and the jumps on High Band and Low Band are significantly correlated with the total number of accelerations and decelerations. The lack of correlation between IMA Jump Count High Band and IMA Accel Medium and IMA Accel High is an interesting result (Table 5).

### 3.5. Interdispositions Explaining the RPE and s-RPE Variable

The effect of the training process on athlete fatigue is a complex and multifaceted phenomenon. Because of that, it was decided to create a set of factors (interdispositions) that would best explain the RPE values after training. We found two External Training Load (ETL) models explaining the RPE variable. In model 1, the strongest explanatory s-RPE variable is the total number of accelerations and the Jump Count High Band. The model explains the variable at the *p* = 0.011 level. The second model includes the variables Accel/Decel, IMA Jump Count High Band, and Mean Total Jumps (Table 6). It is worth noting that the IMA Jump Count High Band parameter fits the models with a negative trend. In contrast, Total IMA Accel, IMA Jump Count Low Band, and Player Load Per Minute are included in the s-RPE explanatory model.

### 3.6. Diagnosed ETL Parameters

Other parameters such as AVG HR (*p* = 0.904); Total Player Load (*p* = 0.944); and Accel/Decel (*p* = 0.207) have no significant association with RPE. Summarizing all observed variables, we find that the number of accelerations shows a significant relationship with the number of jumps (*p* = 0.009). The number of jumps correlates significantly with accelerations at low (*p* = 0.02) and medium levels (*p* = 0.027), and also with the number of decelerations (Sum Decel) (*p* = 0.020).

## 4. Discussion

The foregoing study was aimed at observing and determining the predictors affecting the sensation of fatigue after training, using the RPE and s-RPE index in female volleyball players. The most commonly mentioned variables that may affect s-RPE are jump frequency and the number of jumps [10]. The literature analysis allows us to observe the relationship between s-RPE and the number of jumps during volleyball matches or training. Surprisingly, the mentioned correlations are low [10] or average and opposite to expected [27]. This in turn prompted the authors of the foregoing paper to reflect and attempt to determine the predictors of fatigue in volleyball.

Volleyball is an explosive sport that involves a lot of acceleration and jumping. The results observed confirm the nature of the sport, but surprisingly, the results do not indicate the height and number of jumps, but the number of accelerations during training as a predictor of fatigue in volleyball. The amount of acceleration has a significant relationship with both RPE and s-RPE in observed female volleyball players (Figure 2 and Figure 3). Significant relationships were observed between RPE and s-RPE, between Accel Low and Accel Medium Band, as well as Total Accel acceleration (Table 4). Accelerations in volleyball occur in almost every action, such as movement in the block, defensive play, serving while jumping, and even during the run-up to the attack or serve. Because of that, a commonly considered jump to attack, block, or serve expressed in the number of jumps during a training session may only have an indirect effect on the sensation of fatigue after training. This is confirmed, e.g., by models explaining the RPE and s-RPE ratio (Table 6). In the first model, we observe the number of accelerations and the number of jumps above 40 cm. In the second model, in addition to the Accel/Decel Index, there is the number of jumps in the highest band (High Band) and the Mean Total Jump. It is worth noting that the IMA Jump Count High Band, which enters the model with a negative sign, indicates an inverse correlation (Table 6). Taking a closer look at the IMA Jump Count High Band index, one can see that it only correlates with IMA Accel Low (PC = 0.384) (Table 5). Therefore, the slower the athlete’s run-ups, the higher their jumps are. In jump-based sports, particularly in long jump, it is assumed that the greater the speed of the run-up, the further the jump [28]. Conversely, the biomechanics of jumping in volleyball is very different from long jump or high jump. The lack of association between high run-up velocity and gaining significant jump height in volleyball was confirmed by the studies of Fuchs [29], Ikeda [30], and Wagner [31]. The foregoing paper confirms this. Thus, when considering components of fatigue, the number of jumps can be included, but not the jumps in the highest range (High Band), in case of which 1208 were recorded during the week, and not jumps at low and medium levels, which are related to acceleration below 1.5 m/s^2^ (617 jumps) and acceleration in the range of 1.5–2.5 m/s^2^ (3636 jumps) (Table 3). Model 2 includes an index of the sum of accelerations and decelerations. The Accel/Decel ratio is significantly correlated with the number of jumps and the mean number of jumps. Although the deceleration rate alone was not related to s-RPE, associations can be seen between Accel/Decel and Total Player Load, accelerations, and jumps at all levels (Low, Med., and High Band) (Table 4). It is therefore worthwhile to take a closer look at inhibition values in the context of accurately determining the training load and to look for further relationships between s-RPE and Decel in volleyball.

Three parameters fit into the model explaining the session training load ratio (s-RPE), including Acceleration Count, which directly correlates with s-RPE as the primary factor. This is followed by IMA Jump Count Low Band, which is the only one that shows a relationship with Accel High Band acceleration, and an indicator that includes displacements and jumps per minute. Although the model is statistically significant, the inclusion of player load per minute significantly weakens the strength of the model (Table 6). Such a relationship confirms the importance of acceleration as a major predictor of fatigue in volleyball.

Table 2 shows the analysis of the jumps (SJ, CMJ, CMJA, AJ, BJ), the 20 m run time, and the contact time during the AJ jump. Although only CMJ jump height was statistically significant, the trends of all parameters indicate that the proposed loads were probably optimal for the athletes despite a mean RPE of 4.3. With the proposed intensity, such a training system could be used as a preparatory microcycle in the context of the volleyball season. Due to the small study material, the observed trends would need to be confirmed on a larger number of players. A total of 5488 accelerations and 5506 jumps were performed by the entire team during the training microcycle. In the next step, it would be necessary to determine the optimal parameters of each function on the court in the given age category. 

In conclusion, there are predictors of ET directly related to IT and specifically s-RPE. Such a predictor in volleyball includes the number of accelerations performed during training. The number and frequency of jumps observed so far should be considered an indirect factor of fatigue. The observations made are particularly evident on the second and fifth days of the microcycle, where a large number of jumps were performed, whereas the s-RPE was determined to be small. 

Another question that arises is whether ITL in the form of s-RPE has an actual relationship to player fatigue. The observations in Table 1 suggest that the load was high enough that the speed and jumping values tend to be negative, despite the Borg scale total of 4.3 points.

## 5. Conclusions

Volleyball is a team sport that is mainly associated with jumps. For many years, the number and frequency of jumps and the number of high jumps have been identified as predictors of training load in volleyball. The great amounts of work in low defense positions, moving around the court in action, and even jump run-ups are often overlooked. Therefore, it is common practice for coaches to use defensive improvement training that involves moving the player quickly to the ball as a recovery or maintenance training. It turns out, however, that this form of training can be just as, or even more, taxing than the training associated with completing a sufficient number of jumps.

## Figures and Tables

**Table 1:** Daily training tasks.

Training	Monday	Tuesday	Wednesday	Thursday	Friday
Warm-up	Coordination with balls	General Development	Individual–general development	Strength aspect	Coordination
Technical part	-Defense technique and repetition -Attack-Block	-Pair bounces -Diagonal Attack-Defense	-Setters-Zone IV and II exposition-Defense in function zone -Oblique attack continuity	-Bounces and light attacks over the net in pairs -Reception of the serving -Attack after the reception	-Block -Attack and block -Complex I game
Specialist part	Fragments of a 6 × 6 game	-Complex I by function -Fragment of a Game 6 × 6	-Complex II 6 × 6	Defense and counter-attack-fragment 1 × 1 -Fragment of a Game 6 × 6	-Complex I 6 × 6
Other	-Gym 2 × 10/6rep 60–80%RM -Fitness tests	Non	Non	Gym-10 stages 2 × 8 rep–speed accent	15 min serve technique at the end of the training
Duration	2 h (1 h 07 min volleyball training time)	2 h 15 min 1 h 27 min volleyball training	3 h 15 min 2 h 47 min volleyball training	4 h 3 h 40 min volleyball training	3 h 2 h 17 min volleyball training

Team game organization: complex I (serve reception, setting, and attack), complex II (serve, block/defense, and counterattack).

**Table 2:** Results of motor tests of volleyball female athletes measured before and after the training period. Mean values of variables measured pre- and post-training were compared using *t*-test.

Variable		Mean	SD	CI −95%	CI +95%	SEM	ICC	CA	CV	*t t*-Test	*p t*-Test
SJ	pre	27.25	4.05	24.53	29.96	1.22					
	post	27.70	3.72	25.2	30.2	1.12	0.505	0.671	0.07272727	−0.39	0.705
CMJ	pre	27.59	3.01	25.57	29.61	0.91					
	post	29.54	3.90	26.91	32.16	1.18	0.787	0.881	0.06363636	−2.841	0.018 *
CMJA	pre	32.38	4.59	29.3	35.47	1.38					
	post	33.43	4.89	30.14	36.71	1.47	0.903	0.949	0.03727273	−1.657	0.128
Contact time AJ	pre	0.39	0.05	0.36	0.43	0.02					
	post	0.37	0.04	0.35	0.4	0.01	0.579	0.733	0.05727273	1.759	0.109
AJ	pre	40.41	6.66	35.94	44.88	2.01					
	post	41.64	5.87	37.69	45.58	1.77	0.904	0.949	0.04272727	−1.477	0.17
BJ	pre	31.88	3.90	29.27	34.5	1.18					
	post	32.41	4.82	29.17	35.64	1.45	0.92	0.958	0.02727273	−0.998	0.342
20 m	pre	3.43	0.41	3.16	3.71	0.12					
	post	3.51	0.15	3.41	3.61	0.05	0.486	0.654	0.03454545	−0.861	0.409

Squat Jump (SJ), Countermovement Jump (CMJ), Countermovement Jump with arm swing (CMJA), Spike Jump (AJ), Block Jump (BJ), 20 m sprint (20 m). * Significantly different *p* < 0.05; CI—confidence interval; ICC—interitem correlation coefficient; CA—Cronbach’s alpha reliability coefficient; CV—coefficient of variation; SEM—standard error of the mean; *t*-test—*t*-student test.

**Table 4:** Relationships between RPE and s-RPE and kinematic parameters in the game.

Variable	RPE *p*-Value	Pearson Correlation	s-RPE *p*-Value	Pearson Correlation
Total Player Load	0.004	0.387 **	0.004	0.388 **
AVG Heart Rate	0.081	−0.242	0.267	−0.155
IMA Jump Count Low Band	0.002	0.421 **	0.000	0.497 **
IMA Jump Count Med Band	0.078	0.244	0.043	0.279 *
IMA Jump Count High Band	0.256	0.159	0.872	0.023
Mean Total Jumps	0.019	0.320 *	0.030	0.298 *
Total Jumps Count	0.019	0.320 *	0.030	0.298 *
IMA Accel Low	0.001	0.448 **	0.001	0.456 **
IMA Accel Medium	0.003	0.397 **	0.001	0.453 **
IMA Accel High	0.060	0.258	0.024	0.307 *
Total IMA Accel	0.00	0.480 **	0.000	0.515 **
IMA Decel Low	0.001	0.448 **	0.006	0.370 **
IMA Decel Medium	0.049	0.270 *	0.058	0.259
IMA Decel High	0.49	0.270 *	0.053	0.265
Total Decel	0.001	0.436 **	0.006	0.369 **
ACC/DEC	0.768	0.041	0.238	0.163
AVG Heart Rate	0.081	−0.242	0.267	−0.155
SUM Total Accel; Decel	0.000	0.514 **	0.000	0.486 **
SUM TOTAL JUMP; Accel; Decel	0.001	0.444 **	0.002	0.421 **

* Significant correlation *p* < 0.05. ** significant correlation *p* < 0.01. Total Quality Recovery (TRQ Scale), Rating of Perceived Exertion (RPE), Session Rating of Perceived Exertion (s-RPE), Heart Rate (HR), Inertial Movement Analysis (IMA), Low Band > 20 cm (IMA Jump Count), Med Band 20–40 cm (IMA Jump Count), High Band < 40 cm (IMA Jump Count), acceleration (Accel) and de-acceleration (Decel), IMA Accel/Decel Low > 1.5 m/s^2^, IMA Accel/Decel Med 1.5 m/s^2^–2.5 m/s^2^, IMA Accel/Decel High < 2.5 m/s^2^ training load (TL).

**Table 5:** Comparison of jump count and acceleration and deceleration count in given ranges during volleyball training.

Variable	IMA Jump Count Low Band	IMA Jump Count Med Band	IMA Jump Count High Band	Mean Total Jumps	Total Jumps Count
IMA Accel Low	0.495 **	0.253	0.384 **	0.498 **	0.498 **
IMA Accel Medium	0.547 **	0.371 **	0.278	0.487 **	0.487 **
IMA Accel High	0.522 **	0.069	0.266	0.242	0.242
TOTAL IMA ACCEL	0.576 **	0.274	0.355 *	0.492 **	0.492 **
IMA Decel Low	0.421 **	0.548 **	0.601 **	0.752 *	0.752 *
IMA Decel Medium	0.509 **	0.490 **	0.539 **	0.696 **	0.696 **
IMA Decel High	0.342 *	0.396 **	0.331 *	0.578 **	0.578 **
Total IMA Decel	0.454 **	0.548 **	0.617 **	0.773 **	0.773 **
Accel/Decel	−0.020	−0.359 **	−0.403 **	−0.410 **	−0.410 **

* Significant correlation *p* < 0.05. ** significant correlation *p* < 0.01. Total Quality Recovery (TRQ Scale), Rating of Perceived Exertion (RPE), Session Rating of Perceived Exertion (s-RPE), Heart Rate (HR), Inertial Movement Analysis (IMA), Low Band > 20 cm (IMA Jump Count), Med Band 20–40 cm (IMA Jump Count), High Band < 40 cm (IMA Jump Count), acceleration (ACCEL) and de-acceleration (Decel), IMA Accel/Decel Low > 1.5 m/s^2^, IMA Accel/Decel Med 1.5 m/s^2^–2.5 m/s^2^, IMA Accel/Decel High < 2.5 m/s^2^ training load (TL).

**Table 6:** Multiple regression models for s-RPE.

RPE						
N = 53	b*	Std. Error	b	Std. Error	t(50)	*p*
		of b*		of b		
20 m Free run-up			3.954	0.283	13.980	0.000
IMA Jump Count High Band	−0.383	0.140	−0.015	0.006	−2.741	0.009
TOTAL IMA ACCEL	0.367	0.140	0.009	0.004	2.627	0.011
20 m Free run-up			3.068	0.519	5.915	0
Accel/Decel	0.339	0.143	0.914	0.385	2.376	0.021
IMA Jump Count High Band	−0.393	0.156	−0.016	0.006	−2.513	0.015
Mean Total Jumps	0.456	0.166	0.024	0.009	2.756	0.008
**s-RPE**						
**N = 53**	b*	**Std. Error**	**b**	**Std. Error**	**t(50)**	***p***
		of b*		**of b**		
Free run-up			−85.958	158.896	−0.541	0.591
TOTAL IMA ACCEL	0.359	0.124	2.880	0.995	2.896	0.006
IMA Jump Count Low Band	0.357	0.124	12.741	4.435	2.873	0.006
Player Load Per Minute	0.249	0.111	79.429	35.239	2.254	0.029

Total Quality Recovery (TRQ Scale), Rating of Perceived Exertion (RPE), Session Rating of Perceived Exertion (s-RPE), Heart Rate (HR), Inertial Movement Analysis (IMA), Low Band > 20 cm (IMA Jump Count), Med Band 20–40 cm (IMA Jump Count), High Band < 40 cm (IMA Jump Count), acceleration (ACCEL) and de-acceleration (DECEL), IMA Accel/Decel Low> 1.5 m/s^2^, IMA Accel/Decel Med 1.5 m/s^2^–2.5 m/s^2^, IMA Accel/Decel High < 2.5 m/s^2^ training load (TL).

## Data Availability

The data presented in this study are available on request from the corresponding author.
